# Ethical Considerations Regarding Financial Incentives in Plastic Surgery-Related Health Research

**DOI:** 10.1177/22925503221151185

**Published:** 2023-01-18

**Authors:** Lucas Gallo, Matteo Gallo, Morgan Yuan, Sophocles H. Voineskos, Ronen Avram, Mark H. McRae, Matthew C. McRae, Christopher J. Coroneos, Lisa Schwartz, Achilles Thoma

**Affiliations:** 1 Division of Plastic Surgery, Department of Surgery, 3710 McMaster University, Hamilton, ON, Canada; 2 Faculty of Medicine, University of Ottawa, Ottawa, ON, Canada; 3 Department of Plastic and Reconstructive Surgery, 7938 University of Toronto, Toronto, ON, Canada; 4 Department of Health Research Methods, Evidence, and Impact (HEI), 3710 McMaster University, Hamilton, ON, Canada; 5 Department of Philosophy, 3710 McMaster University, Hamilton, ON, Canada

**Keywords:** ethics, financial compensation, recruitment, éthique, compensation financière, recrutement

## Abstract

**Introduction:** To recruit enough patients to achieve adequate statistical power in clinical research, investigators often rely on financial incentives. The use of these incentives, however, remains controversial as they may cause patients to overlook risks associated with research participation. This concern is amplified in the context of plastic surgery where aesthetic procedures are often more desirable and are not typically covered by public or private insurance plans. Despite this, the ethical debate regarding the use of incentives has largely been absent from plastic surgery journals; therefore, efforts to summarize the existing literature in the context of plastic surgery are necessary. **Methods:** A narrative review of the peer-reviewed published literature was performed to identify existing articles pertaining to financial incentives in plastic surgery-related health research. **Results:** While incentives have the potential to improve sample sizes and promote the recruitment of under-represented patient populations, undue inducement and biased recruitment are possible. At present, there exists a paucity of empirical evidence to substantiate this. Efforts should be taken by investigators and research ethics boards (REBs) to limit the potential negative impacts of monetary compensation. Investigators should place reasonable limits on the value of incentives as well as select models associated with lower risks of undue influence and enrollment bias. When financial remuneration is offered, additional care should be taken by investigators to ensure participants are adequately informed of the risks associated with research participation. **Conclusion:** Current best practice recommendations suggest that proposals submitted to REBs justify the incentives used. Information regarding incentives should also be included within study consent forms and communicated as part of the informed consent process.

## Résumé

**Introduction :** Pour recruter suffisamment de patients pour atteindre une puissance statistique adéquate en recherche clinique, les investigateurs s’appuient souvent sur des incitatifs économiques. Le recours à ces incitations reste néanmoins controversé, car elles peuvent pousser les patients à négliger les risques associés à la participation à la recherche. Cette préoccupation est amplifiée dans le contexte de la chirurgie plastique où les procédures esthétiques sont souvent plus désirables et ne sont habituellement pas couvertes par les régimes d’assurance privés ou publics. En dépit de ces limites, le débat éthique concernant le recours aux incitatifs a été largement absent des journaux de chirurgie plastique. Par conséquent, des efforts visant à résumer les publications existantes sont nécessaires dans le contexte de la chirurgie plastique. **Méthodes :** Une revue narrative de la littérature publiée évaluée par des pairs a été réalisée pour identifier les articles existants relatifs aux incitations financières dans la recherche en santé liée à la chirurgie plastique. **Résultats :** Tandis que les gratifications ont la capacité d’améliorer la taille des échantillons et de promouvoir le recrutement de populations sous-représentées, une incitation indue et un biais de recrutement sont possibles. Il n’y a à l’heure actuelle que peu de données probantes empiriques pour alimenter cette question. Les investigateurs et les comités d’éthique de la recherche doivent néanmoins faire des efforts pour limiter les impacts négatifs potentiels d’une compensation financière. Les chercheurs doivent établir des limites raisonnables à la valeur des gratifications et sélectionner des modèles associés à des risques plus faibles d’influence injustifiée et de biais de recrutement. Une attention supplémentaire doit être donnée par les investigateurs pour s’assurer que les participants sont correctement informés des risques associés à la participation à la recherche quand une rémunération financière est offerte. **Conclusion :** Les recommandations actuelles de meilleures pratiques suggèrent que les projets soumis aux CÉR justifient les gratifications utilisées. Une information concernant les gratifications doit également être incluse dans les formulaires de consentement des études et communiquée dans le cadre du processus d’obtention du consentement éclairé.

## Introduction

Honoraria and financial incentives are frequently used to promote participation in health research.^1^ Although this practice is common, it remains a contentious issue and is the subject of ongoing ethical debate.^2^ Arguments supporting compensation in research have centered around its use as a recruitment tool; however, critics assert that financial incentives may influence participants to disregard the risks associated with research participation.^1–3^

To date, leading ethics guidelines require that informed consent be obtained to participate in research.^3,4^ To meet this standard, subjects should be informed of the purpose, risks, and benefits associated with study enrollment, demonstrate comprehension, and decide voluntarily to participate.^3^ Incentives offered to research participants have been described as unduly influential and therefore may not satisfy the ethical requirement for informed consent.^1^ Despite this concern, existing laws, regulations, and ethical frameworks provide little guidance regarding the use of financial incentives.^2,3^

The 2018 Tri-Council Policy Statement (TCPS) defines incentives as “*anything offered to participants, monetary or otherwise, to encourage participation in research*.”^5^ However, the TCPS does not provide any recommendation regarding what compensation is appropriate; specifically, it “*neither recommends nor discourages the use of incentives*.”^5^ The Belmont Report^6^ and Declaration of Helsinki^7^ also do not address or provide recommendations on this topic.^3^ Given this, research ethics boards (REBs) and investigators are left without specific direction as to whether the use of incentives is ethically appropriate.^2^

While the use of financial incentives continues to be debated within the medical ethics literature, there exists a paucity of discussion assessing the relevance and impact of this issue within plastic surgery. This is in-keeping with the findings of Chung et al^8^ who concluded that, despite the extensive ethical issues faced by plastic surgeons, only a small proportion of the literature was dedicated to discussing ethical principles.^8^ Based on the recommendations in this study, we aim to partially remedy this shortfall by examining the ethical considerations of financial incentives as they relate to those who perform reconstructive (ie, surgery which restores form and function to patients with congenital or acquired abnormalities) or aesthetic (ie, surgery which reshapes normal body structures to improve patient appearance) plastic surgery research.^9,10^ Ultimately, this specialty is unique in that various aesthetic surgeries are not covered by both public and private health insurance plans. Therefore, access to these cost prohibitive procedures through study participation, in addition to other forms of compensation, may be seen as a powerful incentive to participate in research initiatives. Despite these specialty-specific concerns, the ethical debate regarding the use of incentives has largely been absent from plastic surgery journals; therefore, efforts to summarize the existing literature in plastic surgery is warranted.

This article aims to answer the following question: *Is it ethical for clinical subjects to receive incentives to participate in health research?* Justifications for offering incentives and ethical concerns, in addition to rebuttals, are discussed in the context of plastic surgery clinical research.

## Justification for Incentives

Arguments in favor of offering incentives center around their use to: (1) promote overall study recruitment in a timely manner and (2) recruit under-represented and equity-deserving groups.

### Promote Patient Recruitment

The primary justification for the use of incentives in health research is to facilitate the recruitment of research subjects.^1,3,4^ While individuals may choose to enroll in clinical trials for a variety of reasons, studies repeatedly demonstrate that offers of financial compensation significantly increase a prospective individual's likelihood of enrollement.^1,4^

This argument has been substantiated through evaluations of subjects’ willingness to enroll in hypothetical research studies. Specifically, Bentley and Thacker^11^ surveyed 270 university students and analyzed their decision to participate in generic clinical trials at different incentive levels (ie, $350, $800, $1800). The results demonstrated that higher compensation made subjects more willing to participate in research even among trials associated with higher levels of risk. Furthermore, willingness to participate decreased significantly when subjects were asked to enroll without financial compensation. Overall, the authors concluded that incentives made subjects more willing to participate in health research initiatives.^11^ These findings were also in-keeping with the results by Halpern *et al*^12^ who concluded that higher incentives were associated with greater participation interest in hypothetical anti-hypertensive placebo-controlled trials.^12^

Furthermore, the type of incentive model offered is favored to impact participant recruitment.^1,13^ Specifically, incentives can be conceptualized through market, wage-based, reimbursement, or appreciation models—each with associated benefits and disadvantages (Table 1).^1^ In the market model, compensation is determined by supply and demand factors. Given this, the value of proposed incentives is typically increased relative to other models to promote faster recruitment within a given period. Irrespective of the model used, those who support incentives argue that participants who devote time and expose themselves to some level of associated risk deserve to benefit from their participation. Ultimately, this is consistent with the ethical principle that the benefits and risks of research should be distributed fairly within the population.^3,4^

**Table 1.** Incentive Models for Research Participation (Adapted from Grady^1^)

Model	Payment function	Advantages	Disadvantages	Hypothetical surgery examples
Market	Incentive; Supply and demand	Associated with more rapid recruitment Possibility for participants to profit from involvement; little or no financial cost to participation	Higher potential for undue inducement May lead to competition between studies to achieve recruitment goals Different levels of compensation based on location for similar research involvement	Offering interested participants’ aesthetic surgery at a reduced cost if they agree to participate in a trial utilizing a new surgical technique.
Wage	Compensation; Renumeration associated with time commitment (in-keeping with hourly wage for unskilled labor)	Uniform payment of participants for similar work/contributions Low potential for undue inducement	May have negligible impact on participant recruitment Potential to undercompensate some participants and overcompensate others relative to their actual wage	Offering individuals an hourly wage for the duration of their surgery as well as for any follow-up appointments associated with the clinical trial.
Reimbursement	Reimbursement; For expenses associated with study participation	Research participation becomes revenue neutral for participants Low potential for undue inducement	May have negligible impact on participant recruitment Uneven reimbursement between subjects Potential to undercompensate participants if lost wages not reimbursed	Refunding participants for any expenses related to travel, parking, food, childcare, and accommodation associated with their participation in a clinical trial.
Appreciation	Reward; Small tokens of appreciation at the conclusion of study involvement	Expresses gratitude for contributions Low potential for undue inducement	May have negligible impact on participant recruitment	Offering participants’ gift cards/vouchers as tokens of appreciation following participation in a clinical trial.

In plastic surgery research, the implementation of incentives has the potential to address the high prevalence of negative studies that result from inadequate statistical power (ie, low sample sizes). Specifically, Chung et al^14^ concluded that 85% of negative studies with continuous outcomes and 98% of negative studies with dichotomous outcomes had insufficient power to detect a 1 standard deviation difference or 25% change in proportion between interventions, respectively. These results demonstrate that most negative studies within top plastic surgery journals lack the sample size required to detect even moderate-to-large differences between groups. Ultimately, the existing literature supports the use of financial incentives in plastic surgery research to improve willingness to participate, increase sample sizes, and improve statistical power.

While hypothetical study data supports the use of incentives to recruit an adequate number of research subjects, there exists a paucity of empirical data which shows that incentives are necessary in practice.^1^ Specifically, there are many motivations which may prompt individuals to enroll in clinical trials, including curiosity, altruism, or sensation-seeking.^1,15^ Ultimately, given these diverse participant motivations and limited experimental data, one cannot conclude with certainty that incentives are necessary to enroll research subjects in view of their potential for harm (ie, undue inducement, biased enrollment).

### Recruit Hard-to-Reach Subjects

An additional justification for the use of incentives stems from their potential to overcome barriers to research participation in under-represented and equity-deserving groups. Specifically, these individuals may experience a lack of awareness or distrust of health research initiatives given historical ethics abuses.^1,3^ For example, several articles have examined how knowledge of the Tuskegee study, where researchers continued to evaluate the long-term effects of untreated syphilis in African American men despite the availability of antibiotic treatment, has led to distrust and difficult recruitment among minority populations.^16^ Specifically, Shavers et al^17^ noted that participant familiarity with the ethical abuses in the Tuskegee study was associated with distrust of medical researchers in 51% of African Americans and 17% of Caucasians sampled.^17^ Given this finding, the use of incentives may be necessary to overcome distrust among minority groups to achieve racial, ethnic, and social diversity in health research.

This argument has been substantiated through evaluations of subjects’ willingness to participate in hypothetical studies. Specifically, Russell et al^18^ concluded that, “*comments also seemed to suggest it [incentives] was justifiable even when recruitment was not a problem, in order to ensure more equitable recruitment across social strata…*”.^18^ Moreover, a review of the literature performed by Yancey et al^16^ noted that timely participant incentives (ie, monetary compensation) led to improved retention of minority groups in the observational studies and clinical trials analyzed.^16^

In plastic surgery research, current evidence suggests that minority groups are under-represented within clinical trials. Specifically, a systematic review of American plastic surgery randomized controlled trials (RCTs) by Silvestre et al^19^ noted that Hispanic and Asian Americans were 2.3 and 4.0 times less represented, respectively, when compared to the general population of the United States. Ultimately, this study demonstrates limited minority inclusion within plastic surgery RCTs which may impact patient care outcomes.^19^ Given these findings, the use of targeted incentives for minority populations may be warranted within plastic surgery research to address existing racial disparities in the literature and ensure that all populations benefit from future research initiatives. For example, financial reimbursement for travel-related expenses can theoretically be used to enhance minority enrollment in clinical trials where transportation and accessibility present a significant barrier to research participation. In practice, however, there exists a paucity of empirical data to substantiate conclusions that more incentives lead to greater diversity.^1^ In addition, existing theory suggests that promoting ‘trust’ is a complex phenomenon.^20^ Given this, suspicion of the research establishment by minority groups may not be overcome by incentives alone and could possibly be exacerbated by offers of financial compensation.^1,20^ This is in-keeping with the findings by Corbie-Smith et al^21^ who concluded that an “active, community-based recruitment strategy” is necessary to obtain a racially representative study population.^21^

## Ethical Concerns

Ethical concerns associated with the use of financial incentives include the potential for: (1) undue inducement and (2) biased study enrollment.

### Undue Inducement

Concern associated with the use of incentives stem from their potential for undue inducement. In the research context, ‘undue inducement’ refers to an excessive and/or inappropriate reward or offer that may be used to achieve compliance.^3,6^ To address this, the TCPS recommends that “*where incentives are offered to participants, they should not be so large or attractive as to encourage reckless disregard of risks*.”^5^ Specifically, incentives may compromise informed consent by: (1) diminishing one's interest in learning about the risks of research, (2) reducing the voluntary nature of the one's decision to enroll, or (3) compelling individuals to misrepresent themselves to gain entry into a study.^1^

Market incentive models are believed to be at higher risk of undue inducement, relative to reimbursement models, which account for expenses associated with study involvement (eg, parking expenses, meals, travel costs), given the potential for participants to profit from study involvement (Table 1).^1,15^

Furthermore, undue inducement may preferentially impact individuals who rely on research participation as a primary source of income. These professional participants (ie, ‘professional guinea pigs’) may feel obligated to agree to the risks of research, despite deep objections, due to a reliance on incentives for financial support.^4^

Existing evidence supports the notion that incentives may be viewed as unduly influential. Specifically, a survey by Casarett et al^22^ reported that 74.6% of participants felt that a $500 incentive would likely impair their ability to critically consider the risks associated with a hypothetical anti-hypertensive study. Ultimately, this study demonstrates the potential for undue inducement associated with modest and large monetary incentives.^22^ As such, efforts should be made by researchers to mitigate these risks during study design and patient enrollment. For example, in an RCT performed by Thoma et al^23^ comparing Xiaflex injections to palmar fasciectomy in Dupuytren's disease, the authors attempted to mitigate the risk of undue inducement secondary to high cost of Xiaflex treatment by only recruiting participants who had at least 80% insurance coverage prior to study enrollment.^23^ Similar methods can and should be undertaken by all investigators to reduce the potential harms of undue inducement.

In plastic surgery research, enrollment in studies evaluating experimental aesthetic or reconstructive devices may grant individuals access to surgical procedures that are otherwise not covered by public or private health insurance plans. Several online blogs/articles appear to endorse access to free aesthetic surgery through participation in clinical trials.^24,25^ Specifically, access to these cost prohibitive surgeries through study participation may be viewed as a powerful incentive for individuals to enroll in these studies, resulting in potential undue inducement.

For example, a multi-center observational study titled, “Study of the Safety and Effectiveness of Motiva Implants” (NCT03579901) is presently enrolling n=800 participants to undergo breast augmentation.^26^ Ultimately, participants who choose to enroll in this study will receive no-cost primary breast augmentation with experimental silicone gel breast implants in exchange for agreements to attend follow-up visits over 10 years and undergo postoperative magnetic resonance imaging (MRI).^26^ As per 2020 data from the American Society of Plastic Surgery, the average surgeon fee associated with this procedure is valued at $4516 USD.^27^ Given the monetary value associated with this surgery, this study may be viewed as unduly influential, such that participants may be tempted to disregard risks or misrepresent themselves (eg, fail to endorse exclusion criteria or conditions that may impede the use of a MRI) to gain study enrollment, thus warranting ethical concern.

Current evidence suggests that individuals who pursue cosmetic surgery are more likely to be women (89.7%), with a higher prevalence of body dysmorphic disorder and appearance orientation relative to the general population. Therefore, these individuals may be particularly vulnerable to undue inducement associated with incentives of free cosmetic surgery.^28,29^

Despite this argument, there remains no clear definition of what constitutes undue inducement, and there is a paucity of empirical evidence demonstrating that incentives influence participants to disregard risks in practice. Given this, ethical concerns regarding undue inducement may be seen as ‘unwarranted paternalism’, particularly when a study has been reviewed by a REB and risks were determined to be low.^1,3^ Moreover, several studies have demonstrated that offers of financial compensation do not adversely impact participant perceptions of risk.^12,30^ Specifically, individuals were found to spend more time reviewing studies they perceived to be hazardous.^30^ Thus, research subjects may be capable of making informed decisions regarding study participation, despite offers of incentives.

While efforts to limit recruitment incentives may decrease study participation,^31,32^ these efforts are also likely to minimize the potential for undue influence.^1,3,4,30^ Specifically, most investigators and REBs believe that modest incentives are unlikely to obscure risks and/or impair the judgment of most participants.^1,2^ Therefore, risks of undue inducement may be sufficiently minimized if researchers make reasonable efforts to ensure participants adequately understand the potential harms of the study and independently verify inclusion/exclusion criteria at the time of enrollment.^1,4^

In the context of plastic surgery, it may be inappropriate to ask participants to subsidize industry-sponsored research to limit the potential for undue inducement. Specifically, one may argue that participants who devote their time and expose themselves to risks associated with research deserve to benefit from their participation—particularly when there is limited empirical evidence to substantiate concerns for undue inducement and the manufacturer stands to profit significantly from the study findings.

### Biased Enrollment

Incentives may place a disproportionate research burden on low socioeconomic status (SES) or uninsured individuals. Specifically, those with limited financial resources may be more inclined to participate in research as a source of income, relative to wealthier individuals, resulting in biased study enrollment.^1,30^ Moreover, uninsured participants may enroll in research to access treatments that are otherwise unavailable to them—leading to potential therapeutic misconception (ie, confusion between participation in research and receipt of clinical care).^1,4,33^ Given these concerns, the biased enrollment of low SES and uninsured participants may lead to a skewed sample that jeopardizes the external validity of the study. Preferential recruitment would expose low SES individuals to the risks of research while permitting higher SES populations to benefit from the results—this is inconsistent with the ethical principle that the benefits and risks of research should be distributed fairly within the population.^30^ Ideally, by encouraging equitable recruitment practices and stratifying results based on SES, investigators are able to better distribute the costs and benefits of their research.

Despite these concerns, there exists a paucity of empirical evidence demonstrating that financial incentives influence the demographics of research participants. In plastic surgery research, a systematic review by Baxter et al^34^ concluded that health disparities research was limited within the specialty and unevenly distributed across plastic surgery domains—the socioeconomic and demographic data of research participants was not evaluated.^34^ Of note, research without incentives may be seen as too inaccessible to low SES individuals and may bias enrollment in favor of high SES individuals. As higher income is directly associated with improved health outcomes, exclusion of low SES participants would disproportionately bias clinical research data.^1,30^ Furthermore, offers of incentives in health research may enable uninsured individuals to better distinguish between research participation and the receipt of clinical care. Specifically, Grady^1^ concludes that, “*Offering money in return for participation might also enable a patient to say no to the physician instead of feeling obligated to do what the physician suggests*.”^1^ Therefore, financial incentives may decrease the vulnerability of participants and diminish risks of therapeutic misconception.

## Conclusion and Justification

Based on the above considerations, it may be ethical for clinical subjects to receive incentives to participate in health research. In plastic surgery, incentives have the potential to increase sample sizes (ie, improve statistical power) and promote the recruitment of under-represented patient populations. While undue inducement and biased recruitment are possible, at present, there exists a paucity of empirical evidence to substantiate these concerns. However, the absence of this evidence is not sufficient to conclude these ethical concerns do not exist. As such, efforts should be taken by investigators and REBs to limit their potential impact and ensure equitable patient recruitment. Specifically, researchers should place reasonable limits on the value of incentives as well as select models associated with lower risks of undue influence and enrollment bias (Table 1). When incentives are offered, additional care should be taken by investigators to ensure participants are adequately informed of the risks associated with research participation and meet the established study inclusion criteria. Furthermore, investigators should only consider the use of incentives after a study is deemed to be ethically acceptable based on an assessment of its risks and benefits.^1^ Research risks deemed to be unacceptable cannot be made acceptable through offers of incentives.^1^

Current best practice recommendations suggest that proposals submitted to REBs describe the rationale for using incentives, demonstrate how the dollar value was calculated, and provide details on how incentives will be distributed to participants.^1,4^ Information regarding incentives should also be included within study consent forms and communicated to participants as part of the informed consent process.^5^

This analysis has some limitations. Specifically, incentives directed to populations who are particularly vulnerable to exploitation, such as children and individuals unable to provide informed consent as well as a discussion regarding the impact on surgeon incentivization in aesthetic versus reconstructive procedures, warrant specific attention and is beyond the scope of this article. Going forward, future research on this topic should evaluate whether offers of free cosmetic or reconstructive surgery, through participation in plastic surgery research, leads to undue inducement or misrepresentation in practice.
